# Air Pollution Exposure Induces Vascular Injury and Hampers Endothelial Repair by Altering Progenitor and Stem Cells Functionality

**DOI:** 10.3389/fcell.2022.897831

**Published:** 2022-05-27

**Authors:** Alice Costa, Gianandrea Pasquinelli

**Affiliations:** ^1^ Laboratory of Clinical Pathology, Department of Experimental, Diagnostic and Specialty Medicine (DIMES), University of Bologna, Bologna, Italy; ^2^ IRCCS Azienda Ospedaliero Universitaria di Bologna, Bologna, Italy

**Keywords:** air pollution, particulate matter, cardiovascular disease, vascular injury, endothelial repair, endothelial progenitor cells (EPCs)

## Abstract

Extensive evidence indicates an association of air pollution exposure with an increased risk of cardiovascular disease (CVD) development. Fine particulate matter (PM) represents one of the main components of urban pollution, but the mechanisms by which it exerts adverse effects on cardiovascular system remain partially unknown and under investigation. The alteration of endothelial functions and inflammation are among the earliest pathophysiological impacts of environmental exposure on the cardiovascular system and represent critical mediators of PM-induced injury. In this context, endothelial stem/progenitor cells (EPCs) play an important role in vascular homeostasis, endothelial reparative capacity, and vasomotor functionality modulation. Several studies indicate the impairment of EPCs’ vascular reparative capacity due to PM exposure. Since a central source of EPCs is bone marrow (BM), their number and function could be related to the population and functional status of stem cells (SCs) of this district. In this review, we provide an overview of the potential mechanisms by which PM exposure hinders vascular repair by the alteration of progenitor and stem cells’ functionality.

## Graphical Abstract

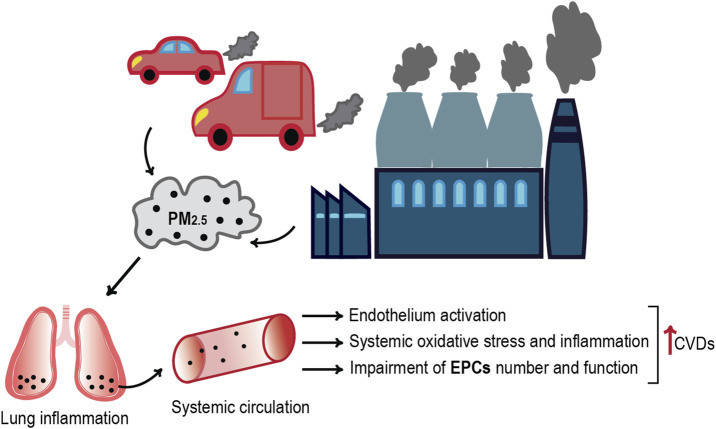

## Introduction

The World Health Organization (WHO) estimates seven million premature deaths worldwide every year due to ambient air pollution (World health statistics, 2021). Extensive epidemiological evidence indicates that not only the respiratory system but almost every organ of the body is affected by polluted air exposure. Indeed, smaller pollutants that arise in the lungs can penetrate the bloodstream reaching the entire body, thus inducing cardiovascular injury, metabolic disorders, systemic inflammation, and carcinogenicity (Franchini and Mannucci, 2011; Rao, 2018). Recent evidence also indicates that air pollution aggravates coronaviruses’ epidemics, such as SARS and COVID-19, with greatest effects in urban areas, where emission levels are highest (Hutter et al., 2020; Cascetta et al., 2021; Salgado et al., 2021).

The outdoor air pollution—especially by PM_2.5_—is deemed to be responsible for about 3.3 million premature deaths (Lelieveld et al., 2015), with accumulating evidence to indicate that the greatest number is related to cardiovascular diseases (CVDs) (Tofler and Muller, 2006; Brook et al., 2010). In Europe, the 45% of the mortality rate is caused by CVDs, which accounts for the 37% within the 28 countries of the European Union (Lelieveld et al., 2015; Wilkins et al., 2017). Furthermore, long-term PM_2.5_ inhalation might result in endothelial injury, vascular homeostasis dysregulation and inflammation, increased coagulation-thrombosis risk, atherosclerosis, and hypertension, effects that could accelerate cardiovascular disease or generate negative cardiovascular events (Cui et al., 2016; Chen et al., 2018; Haberzettl et al., 2018).

In this context of endothelial dysfunction or injury, the endothelial progenitor cells (EPCs) play an essential role, as central regulators of vascular regeneration, angiogenesis, and reendothelialization, which facilitate to vascular repair (Jian et al., 2018; Bayraktutan, 2019). Since the exposure to ambient fine particles leads to cardiovascular injury and, at the same time, defects in vascular repair and regeneration (Chen et al., 2018), it is of common interest to understand how PM_2.5_ affects the number and the functionality of EPCs.

In this review, we summarize the mechanisms by which PM exposure alters EPCs health and how it affects vascular repair following exposure-related cardiovascular injury.

## General Information on PM_2.5_ Air Pollution

In relation to particle size, environmental particulate matter is generally classified in PM_10_ coarse, PM_2.5_, and ultrafine particles (UFPs), characterized by an aerodynamic diameter of 10–2.5 µm, <2.5 µm, and <0.1 µm, respectively (Brook et al., 2010). Urban PM has a varied and complex composition, displaying elemental and organic carbon species (PAHs, nitro-PAHs, alkenes, etc.), especially from a major part of combustion-derived PM. Another important component is the non-carbon one, represented by various minerals’ dusts, sea salt, ammonium, nitrates, and sulfates. Additionally, the surface of PM may accumulate small amounts of biological materials (i.e., endotoxins), which are likely to be involved in airway inflammation and other aspects of the pathophysiological response (Miller and Newby, 2020).

In terms of toxicity, smaller size particles are considered of particular importance for health since they exert the greater effects due to their large reactive surface area and the ability to be inhaled deeply in the alveoli of the lungs (Ohlwein et al., 2019). Among the common fine particle air pollutants, PM_2.5_, containing redox-active transition metals, quinones, and secondary organic aerosols, are mostly generated by the human combustion of fossil fuels (from industry processes, vehicle-exhaust emissions, and power generation) and display the longest atmospheric lifetime (Brook et al., 2010; Lakey et al., 2016). Since not filtered by the respiratory tract, these particles have been proposed to affect the cardiovascular system by reaching the terminal bronchioles via inhalation, entering alveoli, penetrating biological membranes, and inducing an inflammatory response in the lung, with the release of inflammatory mediators that enter the bloodstream (Miller and Newby, 2020).

Among the World’s population, the 91% lives in places where air pollution levels exceed the WHO guidelines limits, set at 5 µg/m^3^ for fine particulate matter PM_2.5_ annual mean (Table 1). The World Health Organization attributes the 58% of outdoor air pollution-related premature deaths to stroke and ischemic heart disease, the 18% due to respiratory disease, and the 6% due to lung cancer (World health statistics, 2021).

**TABLE 1:** Annual mean concentration of fine particulate matter PM_2.5_ by region, population-weighted (Indicator 11.6.2), for the year 2016 (World Health Organization, 2021).

*Region*	*µg/m* ^*3*^
South-East Asia	54
Eastern Mediterranean	51
Africa	39
Western Pacific	39
Europe	13
Americas	12

### PM_2.5_ Impact on Cardiovascular Risk

Several toxicological studies suggest that PM_2.5_ are key mediators of endothelial dysfunction; therefore, this fraction of particulate matter is associated with increased cardiovascular mortality (Brook et al., 2010; O'Toole et al., 2010). Richard B Hayes et al. investigated the relationship between PM_2.5_ exposure and cause-specific cardiovascular disease mortality in 565,477 people, aged 50–70 years, from the National Institutes of Health-AARP Diet and Health Study. They observed an association of 10 µg/m^3^ PM_2.5_ with a 16% and a 14% increase in mortality from ischemic heart disease and stroke, respectively. Moreover, CVD risks were increased with respect to PM_2.5_ exposures in the range of 8–12 µg/m^3^, 12–20 µg/m^3^, and >20 µg/m^3^ (Hayes et al., 2020). Conversely, a study conducted by McGuinn et al. on a cardiac characterization cohort in North Carolina showed no significant effects of PM_2.5_ exposure on biomarkers of cardiovascular risk, but they observed a strong association between these two factors in individuals affected by metabolic syndrome, as hypertension, diabetes, and obesity. Since in the United States the prevalence of the metabolic syndrome is estimated at >30% further studies should not only investigate the PM_2.5_ impact in CVD risk induction but should also consider the effects on those people with pre-existing cardiovascular risk factors (McGuinn et al., 2019).

As already mentioned, a small proportion of particles directly reach the systemic circulation (Brook et al., 2010; Miller and Newby, 2020), where high levels of PM_2.5_ have been correlated with an important decrease in the function of vascular endothelial cells (VECs) that cover the internal surface of blood vessels and are fundamental for the homeostasis of physiological processes (Cao, 2018). Accordingly, endothelial injury increases the release of inflammatory cytokines, leading to blood monocyte recruitment on the activated endothelial monolayer and their infiltration into the sub-endothelial space, essential in the development of atherosclerotic plaques (Lau and Baldus, 2006; Miller and Newby, 2020; Liang et al., 2021).

Several data indicate that PM exposure leads to an increased reactive oxygen species (ROS) production and oxidative stress in the epithelial lining fluid of the human respiratory tracts (Cui et al., 2015a; Lakey et al., 2016). Accordingly, inflammation and vascular-insulin resistance that derived from the increased pulmonary oxidative stress favor cardiovascular disease even with short-term PM_2.5_ exposure (Haberzettl et al., 2018). It is well established that the oxidative stress, pivotal in the pathophysiology of varied CVDs, contributes to endothelial cell activation, which represents an early step in vascular dysfunction development (Horstman et al., 2004; Daiber et al., 2017). In addition, the increased concentration of PM_2.5_ is responsible for VEC induced death, as a result of oxidative stress injury, inflammation, and endoplasmic reticulum stress (Xie et al., 2021). A study conducted by Li et al. on HUVECs (human umbilical vein vascular endothelial cells) exposed to PM_2.5_ revealed an increased ROS generation that resulted in a significant decreased cell viability. Likewise, fine particles’ exposure leads to the reduced activity of superoxide dismutase (SOD) and glutathione peroxidase (GPx), which in turn cause an imbalance in the pro-oxidant/antioxidant homeostasis of HUVECs (Li et al., 2016). In this context, the endothelium inflammation is an inexorable consequence. The study of Montiel-Dávalos et al. on PM_2.5_ toxic effects on HUVECs also reports that the fine particles induce cell activation, followed by monocytic adhesion, through promoting the expression of varied adhesion molecules (E-selectin, P-selectin, ICAM-1) (Montiel-Dávalos et al., 2007), one of the initial events by activated endothelium. In addition, Rui et al. proved that the PM_2.5_-related oxidative stress leads to the expression of ICAM-1 (intercellular adhesion molecule 1) and VCAM-1 (vascular cell adhesion molecule 1) on the cell surface, via the activation of ERK/AKT/NF-kB signaling pathway, thus promoting monocyte adhesion to VECs (Rui et al., 2016). Some evidences also suggest that PM_2.5_-stimulated cytotoxicity in HUVECs accelerates proinflammatory cytokines’ secretion (e.g., CRP, TNF-α, IL-6, and IL-8), leading to endothelial activation through the JAK1/STAT3 signaling pathway stimulation (Hu et al., 2016). In addition, some data showed that PM_2.5_ is responsible for endothelial vasomotor function impairment by increasing the circulating levels of AngII, activating the AT1R (AngII/AngII type 1 receptor) axis, and activating the phospholipase C (PLC) and protein kinase C (PKC) in HUVECs. Accordingly, the biosynthesis of endothelin-1 (ET-1), a protein involved in the cell proliferation and vascular tone regulation, is promoted (Liang et al., 2020). It has also been demonstrated that, in an Ang Ⅱ-induced abdominal aortic aneurysm (AAA) model, PM_2.5_ promotes AAA formation and induces an increase of AAA-related pathological changes, MMP2 and MCP-1 expression in human aortic smooth muscle cells (HASMCs), as the potential result of PM_2.5_-induced senescence and ROS accumulation (Jun et al., 2018).

Furthermore, the *in vivo* study of Chen et al. proved the anti-oxidant and anti-inflammatory effects of probucol, a drug commonly used to lower LDL and HDL cholesterol, in the preservation of the endothelial function by reducing the amount of endogenous nitric oxide (NO) synthase inhibitors. By this way, probucol inhibits the expression of various adhesion molecules, thus promoting the proliferation of endothelial cells, while preventing the apoptosis of the endothelial cells due to oxidative injury (Chen et al., 2018).

## The Regenerative Potential of Endothelial Progenitor Cells in Vascular Repair

Endothelial Progenitor Cells (EPCs) derive from the bone marrow, which are retained via adhesion molecules (i.e., CXCR4) or through interactions between α4β1-integrin and VCAM-1. EPCs originate from a primitive, undifferentiated hematopoietic and vascular cell precursor, circulate in the peripheral blood, and are implicated in neo-angiogenesis (Werner et al., 2005; Singh et al., 2021).

Despite EPCs exact phenotypic identity, origin, and location are debated (Fadini et al., 2012), flow cytometry and immunohistochemistry are the most commonly used techniques for their identification. However, it is difficult to accurately isolate EPCs based on cell surface markers they express because most of them are co-expressed by other endothelial or hematopoietic cells. Thus, the best option for EPCs detection seems the employment of a panel of antibodies targeting markers for hematopoietic cells, immaturity, stemness, and endothelial maturity (CD45, CD133, CD34, and KDR, respectively). In this way, it is possible to better discriminate between endothelial-committed EPCs (CD34 + CD133 + KDR + CD45^−^, CD34 + KDR + CD45^−^, and CD133 + KDR + CD45^−^) and undifferentiated EPCs (CD34 + CD133 + CD45^−^, CD34^+^,CD45-, and CD133 + CD45^−^) (Bayraktutan, 2019).

Consequent to vascular injury, EPCs cells mobilize from the bone marrow into peripheral blood, in response to biochemical stimuli (VEGF, SDF-1α), for example, in the case of ischemic injury (Bayraktutan, 2019; Singh et al., 2021). EPCs are also stimulated to migrate into the circulation upon hypoxic conditions by interactions between CXCR4 and its antagonist SDF-1a or upon matrix metalloproteinase-9 (MMP-9)-mediated release of the soluble c-Kit ligand (Heissig et al., 2002; Petit et al., 2007; Singh et al., 2021). After leaving the bone marrow, EPCs reach the site of endothelial injury where they attach and promote angiogenesis and revascularization by differentiating into mature, functional endothelial cells, or by paracrine stimulation of the existing endothelium to proliferate and migrate (Asahara et al., 1997; Singh et al., 2021). In brief, the EPCs’ mobilization to the vascular injury is influenced by physical factors, such as vascular structure and density, and flow rate, and, also, the expression of cell surface adhesion molecules, in particular VCAM-1 and ICAM-1. Moreover, the increased availability of cytokines, such as IL-6 and GM-CSF (granulocyte-macrophage colony-stimulating factor), contributes to the regulation of EPCs’ counts and behavior, for example, after ischemic injury (Orlic et al., 2001; Fan et al., 2008).

Unsurprisingly, the presence of immature circulating cells in the peripheral blood is considered a marker of organism’s regenerative capacity; therefore, several clinical trials have been designed to increase EPCs’ number at the site of tissue damage (Assmus et al., 2002; Wollert et al., 2004). The EPC-based therapies attract a lot of interest because of the peculiar ability of these cells to detect and repair endothelial damage and to differentiate into other cell lines to promote vasculogenesis, angiogenesis, and neurogenesis after stroke injury (Sekiguchi et al., 2009).

Werner et al. demonstrated the predictive role of CD34 + KDR + circulating EPCs in the occurrence of cardiovascular events and death from cardiovascular causes. They proved that a single measurement of CD34 + KDR + EPCs may help to identify patients with increased cardiovascular risk, predicting cardiovascular outcomes in patients with coronary artery disease. A strongly higher incidence of cardiovascular causes-related death was observed in patients with low baseline levels of EPCs during an observational period of 12 months (Werner et al., 2005).

Several experimental and clinical studies have also shown the EPCs’ involvement in improving revascularization in ischemia models (i.e., hindlimb ischemia and myocardial ischemia), in promoting endothelial health in restenosis and renal failure, and play a role in the pathogenesis of coronary disease, despite in this context their exact role remains to be determined (Werner et al., 2005; Thum et al., 2006; Groleau et al., 2010).

### PM-Exposure Effects on EPCs

Several studies have been performed to evaluate whether and how different factors can influence EPCs’ homeostasis and function. From those studies emerged that EPCs changes are associated with cardiovascular risk factors (diabetes, aging, dyslipidemia, hypertension, atherosclerotic disease, etc.), but also with environmental conditions, such as the exposure to air pollution (Chen et al., 2016; Arcangeli et al., 2017; Singh et al., 2021). In particular, some evidence has indicated that the number and function of EPCs significantly decrease in animals after PM_2.5_ exposure, principally due to the increase of reactive oxygen species (ROS) levels and inflammation (Cui et al., 2016). The study of Cui et al. on C57BL/6 mice exposed to PM for 1 month demonstrated a strong decrease of circulating EPCs due to increased apoptosis via ROS production. Indeed, they observed the high amount of intracellular ROS in circulating EPCs in mice exposed to PM compared to the control group, as the major cause of increased apoptosis of EPCs (Cui et al., 2015b). The findings of variations in EPC number due to air pollution’s redox-active constituents suggest that PM exposure influences EPC levels by inducing oxidative stress.

Another study conducted on both human and mice by O’Toole et al. revealed that high PM_2.5_ level exposure causes a reversible vascular injury, attested by the suppression of EPCs’ number (O'Toole et al., 2010). The same research group also performed a second study in which mice were exposed for nine consecutive days to concentrated ambient PM_2.5_ (CAP), reporting a decrease in the number of circulating EPCs, VEGF-stimulated aortic Akt phosphorylation, and plasma NO levels in *wild-type* mice but not in those overexpressing extracellular superoxide dismutase (ecSOD-Tg). The study also indicates that EPCs from CAP-exposed ecSOD-Tg mice were able to restore hindlimb ischemia when injected post-hindlimb perfusion (Haberzettl et al., 2018). All these findings suggest that PM_2.5_ impairs the vascular endothelial damage repair by altering the abundance and function of EPCs (Liang et al., 2020).

Furthermore, it has been observed that PM exposure not only affects circulating EPCs but also significantly decreases the population of bone marrow stem cells (BMSCs) (Abplanalp et al., 2019), causing a decreased proliferation of these cells via ROS-mediated mechanism in association with Akt pathway inhibition (Cui et al., 2015a). Thus, PM exposure, leading to a decreased number and function of BMSCs, could partially result in an impaired EPCs’ number and function. However, it appears that different EPC subpopulations are differentially affected by PM exposure. Diverse effects based on particles size have been reported in various studies (Table 2), all indicating that EPCs are early, sensitive, and direct targets of air pollution exposure (Liberda et al., 2010; O'Toole et al., 2010; Haberzettl et al., 2012; Niu et al., 2013; Brook et al., 2013; Liberda et al., 2014; DeJarnett et al., 2015; Jantzen et al., 2016; Haberzettl et al., 2018; Li et al., 2021).

**TABLE 2:** Effects of air pollutants on different EPC populations.

*Air Pollutants*	*Effects*	Source
Ni-PM_0,1_	↓ number and function of bone marrow-resident CD34 + KDR + cells	Ref. (Liberda et al., 2010)
Ni-PM_0,1_	↑ number of bone marrow and circulating Sca-1+KDR + CD45^−^ cells	Ref. (Liberda et al., 2014)
CAP	↓ number of circulating Sca-1+KDR + cells	Ref. (Haberzettl et al., 2018)
	↑ number of bone marrow-resident Sca-1+KDR + cells	
PM_2.5_	↓ number of circulating CD31 + CD34 + CD45 + CD133 + cells	Ref. (O'Toole et al., 2010)
	↑ number of bone marrow Sca-1+KDR + cells	Ref. (Haberzettl et al., 2012)
	↓ number of circulating CD34 + KDR+, CD34 + KDR + CD45^−^, CD34 + KDR + CD133 + cells	Ref. (Niu et al., 2013)
Outdoor PM_2.5_ and PM_0.1_	↑ number of circulating CD34 + CD133+ and CD31 + CD133 + cells	Ref. (DeJarnett et al., 2015)
Indoor PM_2.5_ and PM_0.1_	↑ number of circulating CD34 + CD133 + KDR + cells	Ref. (Jantzen et al., 2016)
	↓ number of circulating CD34 + KDR + cells	
PM_10_	↑ number of circulating CD34 + CD133 + cells	Ref. (Brook et al., 2013)

Ni-PM0.1 = nickel nanoparticles; CAP, concentrated ambient PM_2.5_.

Interestingly, sex differences seem to occur in EPC population changes after PM exposure, as demonstrated by Liu et al. in two studies performed on male and female C57BL/6 PM-exposed mice. They observed that PM exposure leads to a decrease of circulating EPCs’ number in male via increased oxidative stress, without affecting the circulating EPCs’ population in females independent of estrogen (Liu et al., 2021). Indeed, PM exposure-induced reduction of circulating and bone marrow’s EPC populations, ascribable to the significant increase of ROS production observed in male, not female, mice, may be due to the decreased expression of SOD1 in male mice (Liu et al., 2022).

Li et al. performed a transcriptomic analysis of cells isolated from CAP-exposed mice, with the aim to identify PM_2.5_-dependent mRNA and miRNA expression changes associated with reduced levels of circulating EPCs and in their proliferation and angiogenic potential defects. They identified, in EPCs derived from CAP-exposed mice, 55 upregulated and 53 downregulated miRNA involved in cell movement, cell death and survival, cellular development, and cell growth and proliferation. In the same group, they also found 122 upregulated and 44 downregulated genes, principally implicated in the regulation of cell movement, cell and tissue development, and cellular assembly and organization. For instance, CXCR3, the receptor for multiple CXC chemokines that promote the mobilization and migration of EPCs, was downregulated in CAP-exposed mice, thus limiting the availability of these cells for the repair of distal tissue damage. Likewise, the downregulation of angiopoietin receptor Tek (Tie2) implies an inefficient EPC maturation in exposed mice. Among the other genes, the proto-oncogene Myc was upregulated, in accordance with the idea that PM_2.5_ may hamper EPC differentiation. Likewise, the upregulation of the pro-inflammatory cytokine Ccl5 suggests a mechanism by which PM_2.5_-exposed EPCs remain in an early, immature state, and thus inefficient in promoting vascular repair. All these results suggest that PM_2.5_-induced changes in gene expression may contribute to EPC dysfunction and such changes may contribute to the adverse cardiovascular outcomes of air pollution exposure (Li et al., 2021).

## Conclusion

Several epidemiological studies positively associate air pollutants exposure with CVDs’ development risk. In particular, the urban PM_2.5_ have been attributed vascular endothelial dysfunction and increased inflammation, which partially promote coagulation-thrombosis and ischemic risk, atherosclerosis, and hypertension, thus promoting and/or accelerating cardiovascular events. PM_2.5_ prevalently exert their effects via oxidative stress and inflammation, and their ability to easily translocate to the blood circulation could account for the widespread effects of these particles around the body; however, the mechanisms remain not completely understood. PM_2.5_ induce VECs’ toxicity and affect their physiological functions, also regulating a set of endothelium-related biomarkers including EPCs. Taken together, the reviewed studies demonstrate that PM_2.5_ exposure leads to EPCs’ dysfunction and altered homeostasis, which in turn contribute to the increased CVDs’ risk. Thus, EPCs’ level and functionality may represent a sensitive biomarker of endothelial injury caused by PM exposure. Furthermore, circulating EPC responses to PM exposure, as well as the responsible mechanistic pathways, may differ between acute and chronic exposures. Indeed, long-term air pollution exposure has been associated with lower levels of EPCs, as a result of cumulative damage to the bone marrow or circulating EPCs, impaired EPCs’ release, and/or decrease of bioavailable EPCs because of their continuous mobilization and subsequent exhaustion. On the contrary, short-term exposure to high doses of PM more likely seems to resemble the biological responses associated with acute EPC levels increase (as myocardial ischemia, exercise, etc.), reflecting the natural mobilization of these cells for endothelial damage repair after PM exposure (Brook et al., 2013). Despite the link between air pollution and EPCs’ dysfunctions is clearly established, the mechanisms whereby different exposure times (long/short-term) could have opposite effects on EPCs’ number and function remain to be fully elucidated. In addition, the possibility that different EPC subpopulations may be differentially affected by PM exposure should be further investigated.

In conclusion, the association between the long-term exposure to ambient fine particles and the risk of cardiovascular disease development implies the need to improve the air pollution abatement for CVD prevention. On the other hand, in addition to good habits (i.e., healthy nutrition, healthy sleeping, and exercise (Kampfrath et al., 2011; Lui et al., 2013; Souza et al., 2020)), new strategies of intervention and prevention are required to improve EPCs’ number and function in order to mitigate the adverse effects of polluted air on human health.
